# Interocclusal Recording for Implant-Supported Fixed Prostheses: Current Materials, Clinical Techniques and Digital Workflows

**DOI:** 10.7759/cureus.117057

**Published:** 2026-09-28

**Authors:** Nezha Ben El Hammi, Iman Ihoum, Abdallah Mouhibi

**Affiliations:** 1 Department of Fixed Prosthodontics, Faculty of Dental Medicine Casablanca, Hassan II University of Casablanca, Casablanca, MAR; 2 Department of Biology and Basic Subjects, Faculty of Dental Medicine Casablanca, Hassan II University of Casablanca, Casablanca, MAR

**Keywords:** dental implants, dental occlusion, implant-supported fixed prosthesis, interocclusal record, verification jig

## Abstract

The occlusal accuracy of an implant-supported fixed prosthesis is largely determined by the quality of its interocclusal record (IOR). Because osseointegrated implants lack a periodontal ligament, occlusal loads are transferred more directly to the prosthetic components and the peri-implant bone, and an imprecise record is thought to contribute to mechanical complications, although this link rests mainly on biomechanical reasoning rather than on direct clinical evidence. Recording the maxillo-mandibular relationship is therefore a critical and technique-sensitive step, especially when extensive edentulous areas deprive the clinician of stable occlusal references. This narrative review analyses the materials and techniques currently available for interocclusal recording in implant-supported fixed prostheses, distinguishing the recording medium, which supports the record intra-orally, from the recording material, which physically registers the relationship. The ideal physical properties expected from both are discussed, namely limited resistance before setting, high dimensional stability over time and sufficient rigidity after setting to ensure a faithful transfer of the occlusion to the working cast. Both the conventional workflow and its digital counterpart, based on intra-oral scanners, scan bodies and reference pins, are considered across the main clinical situations encountered in implant prosthodontics. No single technique is universally applicable; the choice depends on the clinical context, and long-term comparative studies are still required.

## Introduction and background

The long-term success of an implant-supported fixed prosthesis depends largely on careful occlusal management. Unlike natural teeth, osseointegrated implants have no periodontal ligament, so they lack the proprioceptive feedback and physiological mobility that normally buffer and distribute occlusal loads; so, forces are instead transmitted more directly to the prosthetic components and the surrounding bone. This biomechanical difference is thought to predispose to mechanical complications such as ceramic chipping, screw loosening and, more rarely, implant fracture, particularly in bruxers; however, this association is supported mainly by observational data, and a direct causative link between occlusal overload and peri-implant bone loss has not been clearly established [1]. Moreover, no study has directly linked the accuracy of the interocclusal record to the occurrence of these complications. The rationale for precise interocclusal recording therefore rests on biomechanical plausibility and on the practical goal of delivering prostheses that require minimal chairside occlusal adjustment, rather than on direct clinical evidence. Regular occlusal assessment and maintenance nonetheless remain essential [1].

Achieving a physiologically acceptable occlusion also depends on the accuracy of the maxillo-mandibular relationship record [2]. In patients with extensive edentulous spaces, obtaining a correct interocclusal record (IOR) is challenging, because the lack of support for the bite registration material reduces its stability [2]. Several techniques therefore use implant components as supports for the IOR, depending on the available interarch space [2]. These include healing abutments, planning abutments, and impression copings, which are the transfer components screwed onto the implants to record their position. Savabi and Nejatidanesh proposed using the impression copings as a stable intra-oral platform for the IOR, which is then transferred onto the implant analogues to mount the working cast [3]. Zafiropoulos and co-workers developed a workflow based on a transfer key connecting the impression copings, which transfers both the three-dimensional implant position and the maxillo-mandibular relationship for partial fixed prostheses [4].

Digital workflows rely on intra-oral scanners and on two auxiliary components. Scan bodies are geometric markers screwed onto the implants that allow the scanner to locate their position. Reference pins are fixed landmarks used to align successive scans. With these tools, the interocclusal relationship recorded before surgery can be superimposed on the post-operative scan, allowing immediately loaded full-arch interim prostheses to be delivered with a controlled occlusal scheme [5]. Despite this technical evolution, comparative evidence on the accuracy and clinical outcomes of the different recording techniques remains limited, and the optimal strategy is still debated.

This narrative review aims to describe and compare the main materials and techniques used for interocclusal recording in implant-supported fixed prostheses across three clinical situations: single-tooth restorations, short-span fixed dental prostheses and full-arch rehabilitations. The clinical photographs included are original, previously unpublished images used solely to illustrate techniques described in the literature. They are not presented as case reports, and no patient data or clinical outcomes are reported. Written informed consent for their publication was obtained from all patients.

## Review

Methodology

This article is a narrative review of the literature. A non-systematic search of the PubMed/MEDLINE and Scopus databases was conducted and supplemented by a manual search of the reference lists of the retrieved articles. In PubMed, the following search string was used: ("interocclusal record*" OR "jaw relation record*" OR "bite registration" OR "occlusal record*") AND ("dental implant*" OR "implant-supported" OR "implant prosthesis"). The same string was applied to titles, abstracts and keywords in Scopus. The last search was performed in September 2026. No restriction on publication date was applied, so that foundational techniques could be included; only articles published in English were considered.

Articles were eligible if they described a clinical technique, a clinical report, or an in vitro or clinical study addressing the registration of the maxillo-mandibular relationship for implant-supported fixed prostheses, or the physical properties of interocclusal recording materials. Articles dealing exclusively with removable implant-retained overdentures, conference abstracts, and articles without full-text access were excluded. The search identified 567 records (PubMed, n=291; Scopus, n=276). After removal of 41 duplicates, the titles and abstracts of 526 records were screened for relevance, potentially eligible articles were assessed in full text, and the reference lists of the selected articles were hand-searched for additional publications. Selection was agreed upon by all authors. In total, 22 articles were included in this review. Because of the narrative design, no formal risk-of-bias assessment was performed, and the selection process was not intended to be exhaustive or reproducible in the manner of a systematic review; this limitation is discussed below.

Ideal requirements for the interocclusal recording medium and material

For a clinically reliable IOR, two distinct elements have to be considered: the recording medium, which provides intra-oral support for the record, and the recording material, which physically registers the maxillo-mandibular relationship.

The recording medium should offer limited resistance before setting in order to avoid any displacement of the teeth or of the mandible during closure and should become rigid or resilient with minimal dimensional change once set. It should be easy to manipulate, exert no adverse effect on the peri-implant soft tissues involved in the recording procedure, and accurately reproduce the incisal and occlusal surfaces of the opposing teeth [6,7]. In implant prosthodontics, the medium most frequently used is a resin stop attached to the transfer copings, to the framework of the future prosthesis, or to the provisional restoration, depending on the available interarch space [8]. A particular form of this medium is the implant verification jig (IVJ), a rigid resin splint connecting the transfer copings. The IVJ was originally designed to verify the accuracy of the master cast, but it can also serve as a stable support for the interocclusal record [8] (Figure 1).

**Figure 1:**
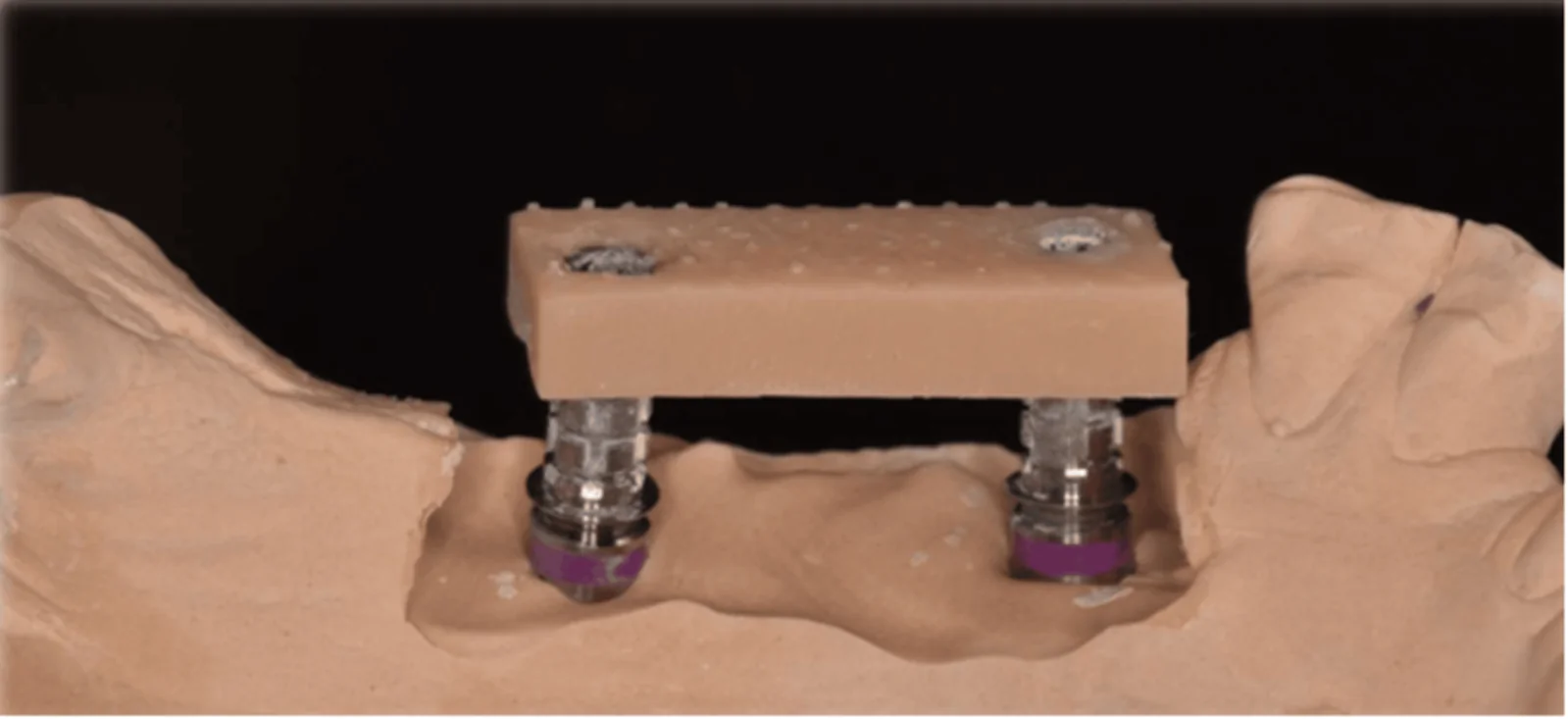
Implant verification jig (IVJ) used as a rigid recording medium for the interocclusal record (laboratory view on the working cast). Two non-engaging transfer copings are splinted together by a sectioned-and-reluted poly(methyl methacrylate) (PMMA) block, providing a rigid, dimensionally stable scaffold for the interocclusal record (technique described by Wong et al. [8]). Original, previously unpublished illustrative photograph.

Three properties of the recording material must be distinguished. Resistance to closure before setting determines whether the material registers the mandibular position without deflecting it. Dimensional stability is the absence of change after setting over time and with variations in temperature and humidity; it determines how long the record remains accurate before mounting. Rigidity, measured as compressive resistance or surface hardness, is the resistance to deformation under load during removal, repositioning on the cast and articulator mounting. These properties are independent: a material may be dimensionally stable yet deform when pressed onto the cast, or rigid yet change dimension over time. The available in vitro studies assessed these properties separately, and their results are reported accordingly (Table 1).

Dimensional Stability

Anup et al. compared polyvinyl siloxane (PVS), zinc oxide-eugenol paste and a bite registration wax (Aluwax) on a die conforming to ADA specification no. 19 (10 specimens per group, measured with a travelling microscope at 1, 24, 48 and 72 hours). PVS was the most dimensionally stable and accurate material [9]. Gosai et al. compared the same wax, a PVS and a bisacryl material (Luxabite, DMG, Hamburg, Germany) over the same four intervals and found that the bisacryl material was the most dimensionally stable at every interval [10]. More recently, Rai et al. reported a different ranking. One PVS showed linear changes of only 0.49%, 0.52% and 0.50% at one hour, 24 hours and one week, and a second PVS showed changes of 1.13% to 1.51%. The bisacryl material expanded at one hour and then contracted, in parallel with weight loss attributed to polymerisation [11]. In that study, specimens were 30 mm in diameter and 2 mm thick, were loaded with 500 g during setting and were measured by stereomicroscopy [11]. In the studies that included them, waxes showed the largest changes, owing to their high coefficient of thermal expansion and to stress release on cooling [9,10].

Rigidity

PVS showed higher surface hardness than wax and zinc oxide-eugenol [9], whereas the bisacryl material was harder than both PVS and wax at 24 hours [10]. In compression testing according to ADA specifications, the bisacryl material showed the lowest compressive strain (0.48%, displacement 0.099 mm). A thermoplastic resin followed (0.60%, 0.123 mm), and a PVS bite registration material was the least resistant [12].

Overall, these data show that the ranking between PVS and bisacryl depends on the product and the testing protocol, whereas the poor performance of waxes is consistent across studies. Because the protocols are heterogeneous and the specimens are standardised laboratory samples, the clinical relevance of these differences, typically fractions of a percent, has not been established. The data nevertheless consistently support mounting the casts shortly after recording [11].

**Table 1:** Properties of interocclusal recording materials reported in in vitro studies, with their implications for implant-supported fixed prostheses PVS: Polyvinyl siloxane; IOR: interocclusal record.

Material	Resistance before setting	Dimensional stability (conditions)	Rigidity after setting	Suggested use in implant IOR	Main limitations
Bite registration wax (e.g., Aluwax)	Variable; depends on softening temperature	Least stable in all studies (1-72 h) [9,10]	Lowest surface hardness [9,10]	Diagnostic or provisional records only	Thermal sensitivity, stress release; requires immediate mounting
PVS bite registration material / putty	Low (registration grade) to moderate (putty)	Most stable in [9]; 0.49-1.51% linear change over 1 h-1 week, product-dependent [11]	Harder than wax [9]; least resistant to compression in [12]	Full-arch records on impression copings [3]	Can deform under pressure when thin; product variability
Bisacryl material (e.g., Luxabite)	Low	Most stable in [10]; early expansion then contraction in (N1)	Highest hardness [10]; lowest compressive strain, 0.48% [12]	Records requiring high rigidity before mounting	Brittle in thin sections; conflicting stability data
Light-curing composite resin	Low; sets on command	No IOR-specific data identified	High (by material class)	Small increments on framework, jig or copings	Polymerisation shrinkage; use based on handling properties

Recording techniques according to the clinical situation

The clinical workflow used to register the maxillo-mandibular relationship varies according to the extent of edentulism and to the prosthetic design. Three main clinical situations can be distinguished: single-tooth implant restorations, short-span implant-supported fixed dental prostheses (FDPs), and full-arch implant-supported restoration, each requiring an adapted choice of recording medium and material [2-4].

The Single-Tooth Implant Restoration

When a single implant is restored within a complete or partially dentate arch, the surrounding natural dentition can serve as an occlusal reference. The diagnostic casts can be hand-articulated in maximum intercuspation, and the occlusion should be carefully controlled to improve clinical success [13]. As summarised in the review by Yuan and Sukotjo, osseointegrated implants exhibit reduced axial mobility (3-5 µm) compared with natural teeth (25-100 µm), which makes them more susceptible to bending loads and to mechanical complications such as screw loosening, veneer fracture or abutment fracture [13]. To protect the implant from overload, the occlusal scheme should follow the principle of light contact in maximum intercuspation, with excursive guidance provided by the natural dentition and no working or non-working interferences on the implant restoration [13]. Implant-supported prostheses should also be monitored at follow-up, as occlusal forces and contact times have been shown to increase significantly over a three-year period, a change commonly attributed to continued eruption of the opposing teeth and to occlusal wear [14].

When occlusal refinement is required at the framework stage, a small increment of a rigid, dimensionally stable recording material can be added to the occlusal surface of the framework. The patient then closes into maximum intercuspation, and the material is allowed to set in this position (Figure 2), transferring the position of the opposing contacts to the laboratory. The record is then repositioned on the working cast so that the laboratory can verify the occlusal scheme before veneering (Figure 3). A light-curing composite resin is suitable for this purpose because it sets on command and is rigid after polymerisation; however, its use relies on these handling properties, as no in vitro study specific to interocclusal recording was identified for this material.

**Figure 2:**
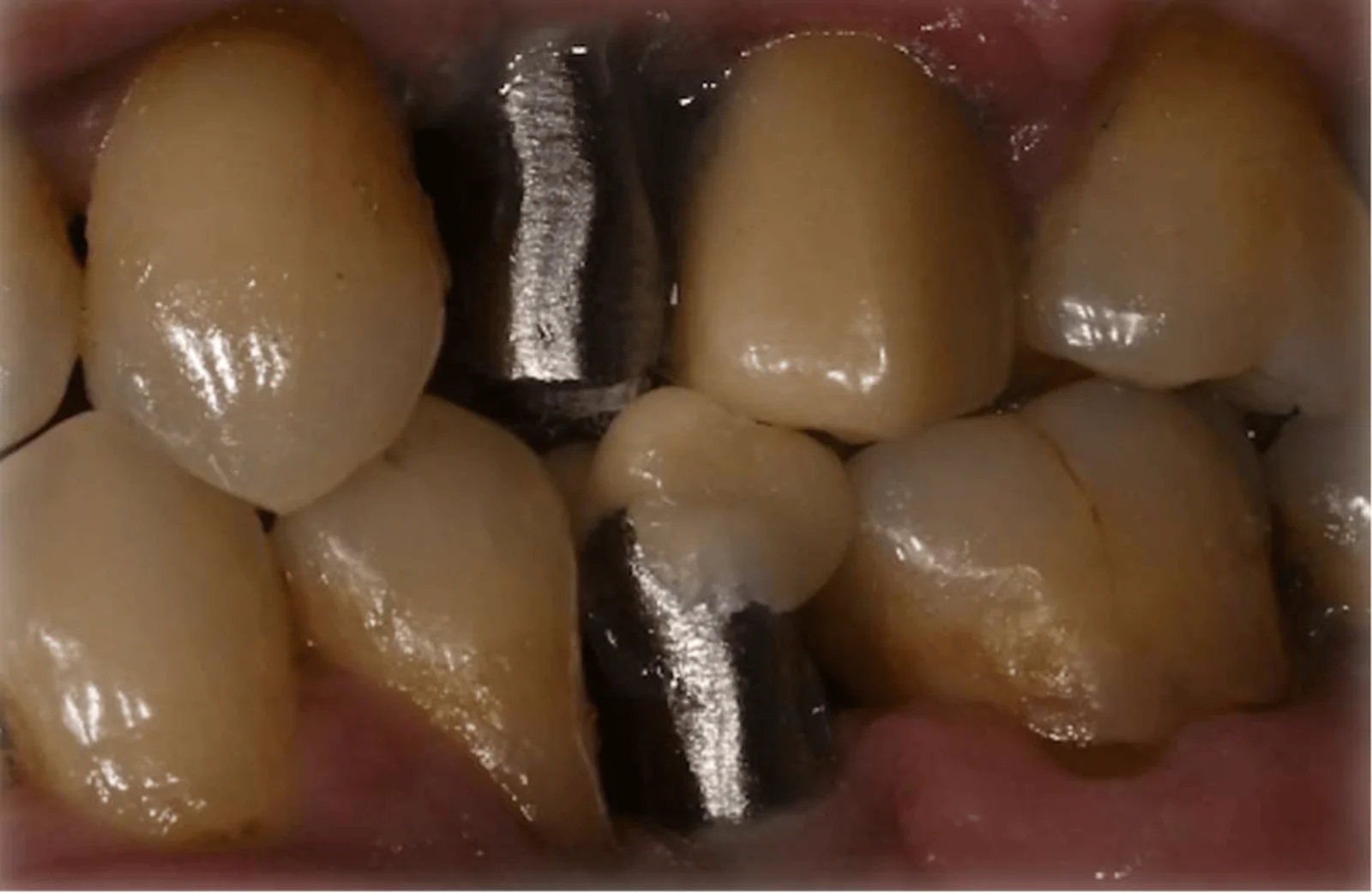
Single-tooth implant restoration: Chair-side interocclusal recording using the prosthetic framework as recording medium and a light-curing composite resin as recording material. Intra-oral view in maximum intercuspation, showing the framework with the polymerised composite stop registering the antagonist occlusal contacts. Original, previously unpublished illustrative photograph; written informed consent for publication was obtained from the patient.

**Figure 3:**
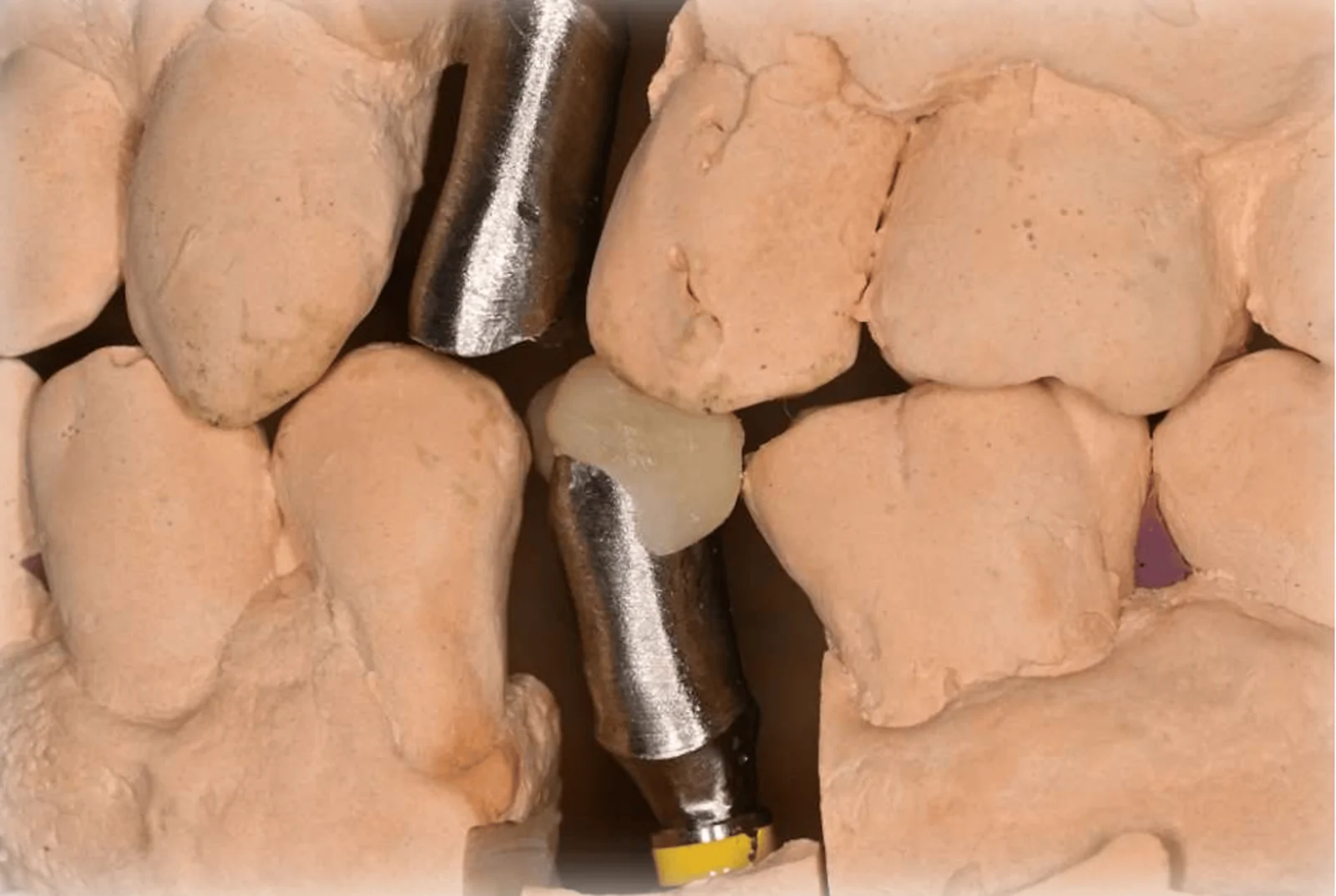
Single-tooth implant restoration: Interocclusal record transferred onto the working cast, allowing the laboratory to verify the occlusal scheme before veneering. Original, previously unpublished illustrative photograph; written informed consent for publication was obtained from the patient.

The Short-Span Implant-Supported Fixed Dental Prosthesis

In short, edentulous spans rehabilitated with implant-supported FDPs, the recording procedure must compensate for the absence of opposing dental contacts in the edentulous area while still benefiting from the stable occlusion of the adjacent natural teeth. A reliable method is to use a sectioned-and-reluted resin wedge, that is, a rigid PMMA or autopolymerising acrylic block, splinted intra-orally to the impression copings or to the temporary cylinders of the implants [8]. The procedure can be summarised as follows (Figures 4, 5): (i) a rigid resin wedge is splinted intra-orally to the transfer copings; (ii) a small increment of light-curing composite is applied to its occlusal surface; (iii) the patient closes into maximum intercuspation against the antagonist arch; (iv) the composite is light-cured in occlusion, and the record is checked after reopening; and (v) the numbered wedges are transferred onto the working cast for articulator mounting [8]. In this configuration, the resin wedge primarily serves to verify implant position and also acts as a stable support for the record, while the composite acts as the recording material.

**Figure 4:**
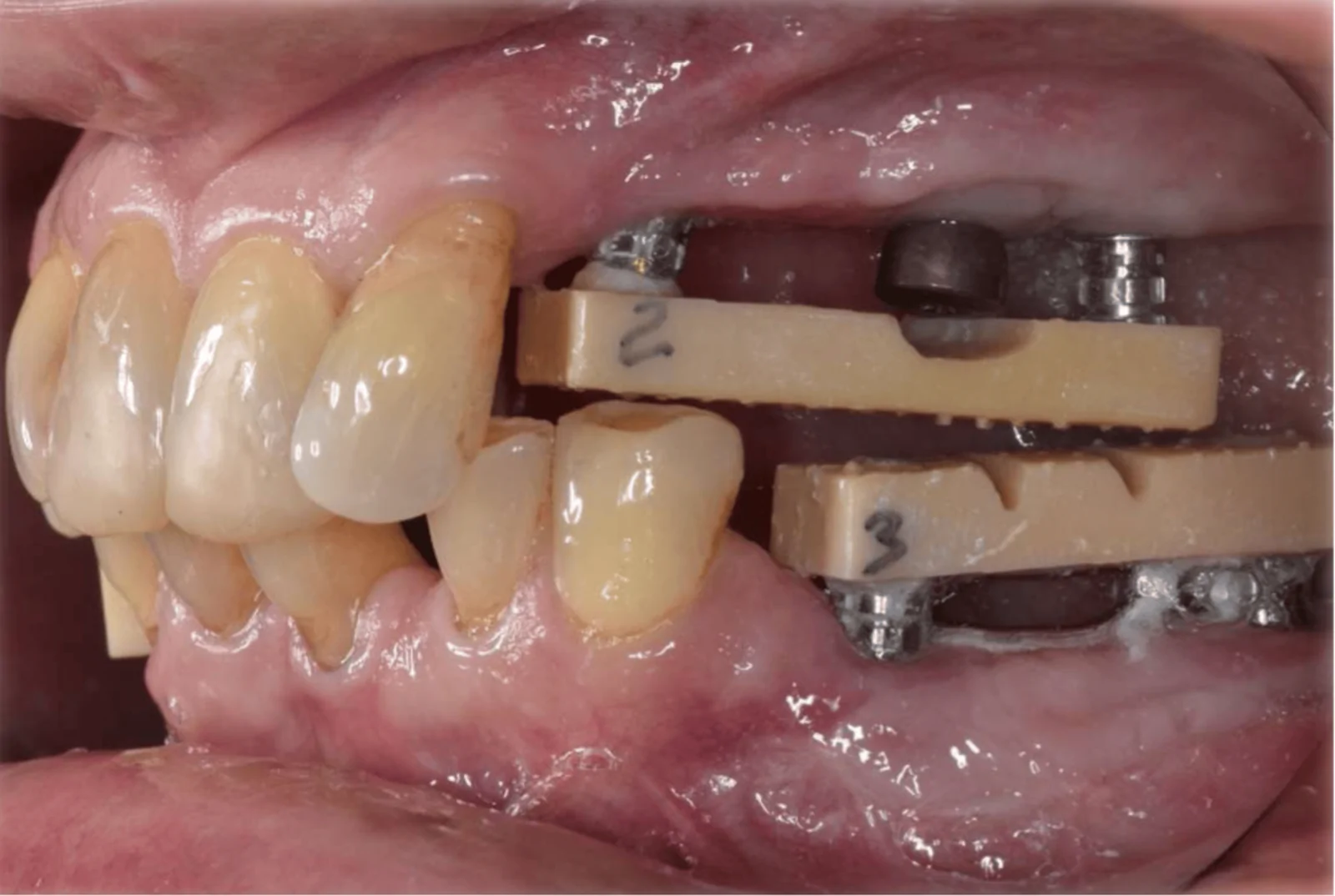
Recording of the maxillo-mandibular relationship on a short-span implant-supported fixed dental prosthesis using rigid resin wedges as the recording medium. Intra-oral registration in maximum intercuspation against the residual natural dentition. Original, previously unpublished illustrative photograph; written informed consent for publication was obtained from the patient.

**Figure 5:**
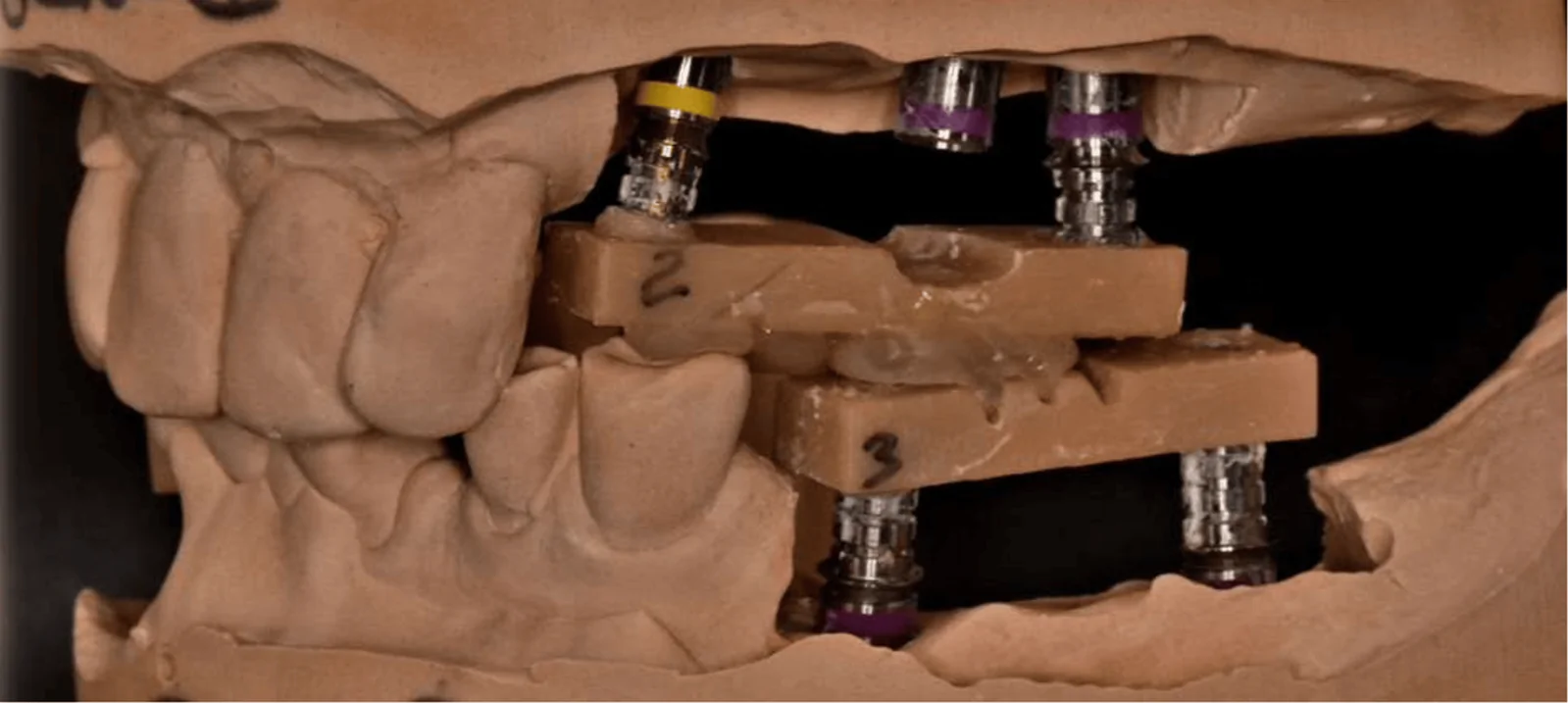
Recording of the maxillo-mandibular relationship on a short-span implant-supported fixed dental prosthesis using rigid resin wedges as the recording medium. Transfer of the numbered wedges onto the working cast for articulator mounting and prosthesis design. Original, previously unpublished illustrative photograph; written informed consent for publication was obtained from the patient.

The Full-Arch Implant-Supported Restoration

In edentulous patients, the absence of natural occlusal references limits the ability to establish centric relation or to determine the vertical dimension of occlusion (VDO), making interocclusal recording particularly challenging [15]. This situation is illustrated by a maxillary edentulous arch opposing a mandibular Kennedy Class I arch (Figure 6). In such cases, a set of provisional prostheses is fabricated beforehand and used clinically to validate the VDO, the centric relation and the aesthetic parameters before the definitive registration is performed [16]. The technique described by Savabi and Nejatidanesh, whose principle of using implant components as a support for the record has been developed in later full-arch protocols [15] and compared in vitro with other implant bite registration techniques [17], can be summarised as follows [3]. A definitive impression of the implants is first made with a custom tray and an elastomeric impression material. The impression copings are then screwed onto the implants, and any portion that interferes with complete closure in the maximum intercuspation position is precisely trimmed away with a sharp scalpel to allow unrestricted intermaxillary closure during the registration. The interocclusal record is subsequently made with a putty-type PVS material applied over the shoulder of the impression copings, which provides a wide and stable supporting platform for the recording material [3]. After setting, the impression copings and the interocclusal record are repositioned together on the implant analogues of the definitive cast, and the mandibular cast is mounted on the articulator using the record as a mounting guide [3].

**Figure 6:**
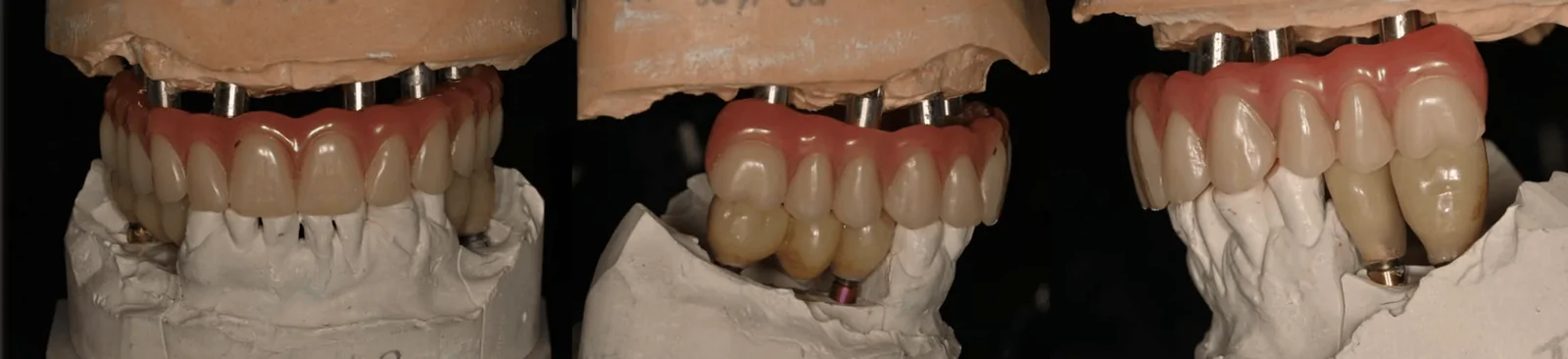
Verification of the occlusal scheme on the articulator after mounting based on the putty vinyl polysiloxane interocclusal record. Direct comparisons between recording workflows remain scarce. In a randomised controlled clinical trial of 20 patients receiving complete-arch implant-supported fixed prostheses, conventional and intra-oral scanner-based maxillary occlusal records produced a similar distribution and number of occlusal contacts, in both the anterior (p=0.439) and posterior (p=0.227) regions [18]. The digital record required less occlusal and clinical adjustment time but more laboratory and total workflow time [18]. No other clinical study directly comparing full-arch interocclusal recording techniques was identified. Original, previously unpublished illustrative photograph; written informed consent for publication was obtained from the patient.

In this configuration, the recording medium is therefore the combination of the provisional prosthesis, which transfers the vertical dimension and the centric occlusion previously accepted by the patient, and the impression copings, which provide a rigid and repositionable platform on the implants. The recording material is a high-rigidity PVS putty, chosen for its low pre-set viscosity, its high dimensional stability over time and its high rigidity after setting, which together ensure a faithful transfer of the registered occlusion to the working cast [3,6,7]. The articulator mounting allows the centric occlusion and the eccentric movements of the future prosthesis to be checked before clinical verification at delivery (Figure 6).

Digital workflows for interocclusal recording

In digital workflows, intra-oral scanners (IOS), scan bodies and reference pins replace the physical impression and the elastomeric record. Two distinct sources of error must be considered: the accuracy of the digital implant impression, and the accuracy of the virtual interocclusal record that articulates the two arches. As with analogue techniques, both depend on the number of stable references available [19,20].

Impression Accuracy

In a systematic review of in vivo studies, deviations of 80.7 and 118.9 µm were reported at teeth adjacent to a single implant. In partially edentulous arches, reported trueness ranged widely, from 27.4±13.5 µm to 360±46 µm; in the study reporting the latter value, conventional pick-up impressions showed 160±25 µm [20]. In edentulous arches, mean deviations of 226 µm (range 21-638 µm) were reported in mandibles scanned without auxiliary devices, and four of 25 scans could not be completed. In maxillae scanned with palatal fiducial markers, the deviation was 162±77 µm [20]. These values should be interpreted against the proposed thresholds for acceptable misfit, which range from 100 to 150 µm [20]. The precision of single-implant scans ranged from 57.2 to 176.7 µm depending on the scanner, compared with 32.7 µm for conventional impressions [20]. Because of the heterogeneity of measurement methods, the review concluded that the trueness and precision of IOS in partial and complete arches remain unclear [20].

For complete arches, stereophotogrammetry (SPG) has been proposed as an alternative to IOS. A 2025 meta-analysis of 13 studies (three in vivo, 10 in vitro) found that SPG was more accurate than IOS in 10 of the 13 studies. Mean angular trueness ranged from 0.24° to 0.80° with SPG and from 0.28° to 1.74° with IOS, the latter exceeding the 1° threshold considered necessary for passive fit. Mean surface trueness ranged from 5.18 to 48.74 µm with SPG and from 14.8 to 67.72 µm with IOS [21]. The authors nevertheless recommended a rigid prototype try-in until further clinical trials are available [21].

Virtual Interocclusal Record

When natural teeth are present, a buccal scan in maximum intercuspation provides a well-referenced record, and virtual articulation has been reported to be accurate [19]. In complete arches, reference pins and the superimposition of a pre-operative scan onto the post-operative scan allow the VDO, occlusal plane and intermaxillary relationship to be transferred, enabling immediately loaded interim prostheses without intra-operative impressions [5,22]. Clinical comparative data remain limited to a single randomised trial [18], and the long-term performance of digitally recorded occlusion still requires prospective validation.

Conventional Versus Digital Approaches According to the Clinical Situation

In single-tooth restorations, the conventional approach uses the framework as the recording medium and a light-curing composite as the recording material. Its digital counterpart, a buccal scan in maximum intercuspation, benefits from abundant dental references and represents the most predictable digital scenario [19,20]. In short-span fixed dental prostheses, a rigid resin wedge or verification jig splinted to the transfer copings can be replaced by a buccal scan supported by the adjacent teeth [8,19]. However, the accuracy of the digital impression varies widely in this situation and depends on the scanning strategy and on inter-implant angulation [20]. In full-arch restorations, the conventional approach combines a validated provisional prosthesis, impression copings and a rigid PVS putty [3]. The digital approach relies on scan bodies, reference pins, the superimposition of pre- and post-operative scans, and possibly photogrammetry [5,21,22]. It yields a similar distribution of occlusal contacts in the only available randomised trial, but at the cost of longer laboratory time [18], and comparative clinical evidence remains limited.

Limitations

This review has several limitations. Its narrative, non-systematic design, without formal quality appraisal of the included studies, exposes it to selection bias in the choice of references. The evidence on recording materials derives from a small number of in vitro studies with heterogeneous protocols and partly conflicting results, whose clinical relevance is uncertain. Most clinical techniques are supported only by technical descriptions, and a single randomised trial has directly compared full-arch recording workflows. The digital accuracy data mainly concern implant impressions rather than the interocclusal record itself. Finally, no study has linked the accuracy of the interocclusal record to clinical outcomes, so the importance attributed to recording precision rests largely on biomechanical reasoning. The clinical photographs are illustrative and do not constitute outcome data.

Future directions

Future research should include standardised in vitro protocols that report dimensional stability and rigidity separately, clinical trials comparing conventional, intra-oral scanning and photogrammetric workflows for each clinical situation, and prospective studies relating the accuracy of the interocclusal record to occlusal adjustment time and to mechanical complications.

## Conclusions

The IOR is a critical step in the fabrication of implant-supported fixed prostheses, although its influence on clinical outcomes has not been directly demonstrated. The recording strategy should be adapted to the clinical situation. In single-tooth restorations, the framework can act as the recording medium, supported by the residual dentition. In short-span prostheses, a rigid resin wedge splinted to the transfer copings stabilises the record. In full-arch restorations, a validated provisional prosthesis combined with impression copings and a rigid PVS putty remains the most documented analogue approach. In vitro evidence consistently discourages the use of waxes, whereas the ranking between PVS and bisacryl materials depends on the product and protocol; whatever the material, casts should be mounted promptly. Digital workflows are a promising alternative, particularly for immediately loaded full-arch rehabilitations, and photogrammetry appears more accurate than intra-oral scanning for complete-arch implant impressions, but comparative clinical evidence remains limited.

## References

[1] Stilwell C (2024). Occlusal considerations in maintaining health of implants and their restorations. Br Dent J.

[2] Monzavi A, Alikhasi M, Taghavi F (2012). Occlusal recording components for dental implant-supported prostheses. J Dent (Tehran).

[3] Savabi O, Nejatidanesh F (2004). Interocclusal record for fixed implant-supported prosthesis. J Prosthet Dent.

[4] Zafiropoulos GG, Hoffmann O, Deli G (2014). A method for fabrication of implant-supported fixed partial dentures. J Oral Implantol.

[5] Espona J, Roig E, Ali A, Roig M (2020). Immediately loaded interim complete-arch implant-supported fixed dental prostheses fabricated with a completely digital workflow: A clinical technique. J Prosthet Dent.

[6] Arjun Sl, Manoharan Ps, Eugene R, Anandapandian PA (2024). Dimensional Stability and Compressive Resistance of Three Interocclusal Record Materials: A Comparative In Vitro Study. Cureus.

[7] Chandu GS, Khan MF, Mishra SK, Asnani P (2015). Evaluation and comparison of resistance to compression of various interocclusal recording media: an in vitro study. J Int Oral Health.

[8] Wong AW, Nedelcu R, Hamilton A (2025). Clinical recommendations for implant verification jigs in analogue and digital workflows. A narrative review. Aust Dent J.

[9] Anup G, Ahila SC, Vasanthakumar M (2011). Evaluation of dimensional stability, accuracy and surface hardness of interocclusal recording materials at various time intervals: an in vitro study. J Indian Prosthodont Soc.

[10] N Gosai K, B Patel I, Poonia P, Sutaria B, Trivedi M, Mori S (2024). Dimensional stability and surface hardness of inter-occlusal recording materials. Bioinformation.

[11] Rai V, Aleemuddin M, Kulasekaran K, Pai SA, Singh A (2025). In vitro evaluation of time-dependent linear dimensional changes in four interocclusal recording materials. Cureus.

[12] Koppolu SK, Manoharan PS, Rajkumar E, Ezhumalai G (2023). Evaluation of dimensional stability, compressive resistance, and detail reproduction of thermoplastic resin ((BD)IMPRESS), elastomeric and composite bite registration material: an in vitro study. J Indian Prosthodont Soc.

[13] Yuan JC, Sukotjo C (2013). Occlusion for implant-supported fixed dental prostheses in partially edentulous patients: a literature review and current concepts. J Periodontal Implant Sci.

[14] Luo Q, Ding Q, Zhang L, Xie Q (2020). Analyzing the occlusion variation of single posterior implant-supported fixed prostheses by using the T-scan system: a prospective 3-year follow-up study. J Prosthet Dent.

[15] Zafiropoulos GG, Galil AA, Deli G (2014). An interocclusal recording method for the fabrication of full-arch implant-retained restorations. J Oral Implantol.

[16] Misch CM (2004). Immediate loading of definitive implants in the edentulous mandible using a fixed provisional prosthesis: the denture conversion technique. J Oral Maxillofac Surg.

[17] Park DH, Park JM, Choi JW (2017). Accuracy of several implant bite registration techniques: an in-vitro pilot study. J Adv Prosthodont.

[18] Pereira AL, Campos MF, Torres AC, Carreiro AD (2024). Conventional and digital maxillary occlusal record for the manufacture of complete-arch implant-supported fixed prostheses: randomized controlled clinical trial. Clin Oral Investig.

[19] Abduo J, Rasaie V (2025). Digital workflows in prosthodontics. Aust Dent J.

[20] Ma J, Zhang B, Song H, Wu D, Song T (2023). Accuracy of digital implant impressions obtained using intraoral scanners: a systematic review and meta-analysis of in vivo studies. Int J Implant Dent.

[21] Pozzi A, Arcuri L, Carosi P, Laureti A, Londono J, Wang HL (2025). Photogrammetry versus intraoral scanning in complete-arch digital implant impression: a systematic review and meta-analysis. Clin Implant Dent Relat Res.

[22] Lorenzetti M, Lorenzetti V, Carossa M, Cavagnetto D, Mussano F (2021). Using a preoperative scan digital impression and a digital index to build immediate interim full-arch implant-supported prosthesis: a case report and proof of concept. Appl Sci.

